# Pulmonary epithelial barrier and immunological functions at birth and in early life - key determinants of the development of asthma?  A description of the protocol for the Breathing Together study

**DOI:** 10.12688/wellcomeopenres.14489.1

**Published:** 2018-05-17

**Authors:** Steve Turner, Adnan Custovic, Peter Ghazal, Jonathan Grigg, Mindy Gore, John Henderson, Clare M. Lloyd, Ben Marsland, Ultan F. Power, Graham Roberts, Sejal Saglani, Jurgen Schwarze, Michael Shields, Andrew Bush

**Affiliations:** 1 Child Health, University of Aberdeen, Aberdeen, AB25 2ZG, UK; 2 Department of Paediatrics, Imperial College and Royal Brompton Hospital, London, SW3 6NP, UK; 3 Division of Infection and Pathway Medicine, Deanery of Biomedical Sciences, University of Edinburgh Medical School, Edinburgh, EH16 4TJ, UK; 4 Centre for Child Health, Blizard Institute, Queen Mary University of London, London, E1 2AT, UK; 5 Population Health Sciences, Bristol Medical School, University of Bristol, Bristol, BS8 1TH, UK; 6 Faculty of Medicine, National Heart & Lung Institute, Imperial College London, London, SW7 2AZ, UK; 7 Department of Immunology and Pathology, Monash University, Melbourne, VIC, 3004 , Australia; 8 Centre for Experimental Medicine, School of Medicine, Dentistry and Biomedical Sciences, Queen’s University Belfast, Belfast, BT9 7BL, UK; 9 Clinical and Experimental Sciences and Human Development and Health, Faculty of Medicine, University of Southampton, Southampton, SO17 1BJ, UK; 10 NIHR Southampton Respiratory Biomedical Research Unit, University Hospital Southampton NHS Foundation Trust, Southampton, SO16 6YD, UK; 11 The David Hide Asthma and Allergy Research Centre, St Mary’s Hospital, Newport, Isle of Wight, PO30 5TG, UK; 12 Child Life and Health and MRC-Centre for Inflammation Research, Queen's Medical Research Institute, University of Edinburgh, Edinburgh, EH9 1UW, UK

**Keywords:** Asthma, Child, Epithelial cell, Genetics, Infant, Longitudinal studies, Lymphocyte, Microbiome, Ribonucleic acid

## Abstract

**Background.**  Childhood asthma is a common complex condition whose aetiology is thought to involve gene-environment interactions in early life occurring at the airway epithelium, associated with immune dysmaturation.  It is not clear if abnormal airway epithelium cell (AEC) and cellular immune system functions associated with asthma are primary or secondary.  To explore this, we will (i) recruit a birth cohort and observe the evolution of respiratory symptoms; (ii) recruit children with and without asthma symptoms; and (iii) use existing data from children in established STELAR birth cohorts.    Novel pathways identified in the birth cohort will be sought in the children with established disease.  Our over-arching hypothesis is that epithelium function is abnormal at birth in babies who subsequently develop asthma and progression is driven by abnormal interactions between the epithelium, genetic factors, the developing immune system, and the microbiome in the first years of life.

**Methods.**  One thousand babies will be recruited and nasal AEC collected at 5-10 days after birth for culture.  Transcriptomes in AEC and blood leukocytes and the upper airway microbiome will be determined in babies and again at one and three years of age. In a subset of 100 individuals, AEC transcriptomes and microbiomes will also be assessed at three and six months.  Individuals will be assigned a wheeze category at age three years.  In a cross sectional study, 300 asthmatic and healthy children aged 1 to 16 years will have nasal and bronchial AEC collected for culture and transcriptome analysis, leukocyte transcriptome analysis, and upper and lower airway microbiomes ascertained.  Genetic variants associated with asthma symptoms will be confirmed in the STELAR cohorts.

**Conclusions.**  This study is the first to comprehensively study the temporal relationship between aberrant AEC and immune cell function and asthma symptoms in the context of early gene-microbiome interactions.

## Introduction

Asthma is the most common non-communicable disease of childhood and affects 1 million children in the UK ^1^. A child is admitted to hospital for a severe asthma attack every 20 minutes in the UK ^1^, with many more seeking unscheduled healthcare assistance with less severe attacks. Regular apparently “minor” asthma symptoms can affect the child’s sleep, exercise, school attendance and mental wellbeing. The NHS budget for asthma treatment is approximately £1 billion ^1^ with approximately 25% of this being spent on children, and one in twenty school-aged children are prescribed asthma preventer treatment. Childhood asthma is a life-long burden to the individual since symptoms often persist into adulthood. Asthma is associated with early poor lung function, which persists into adult life, leading to increased risk of chronic obstructive pulmonary disease ^2, 3^, which is the fourth most common cause of death in the UK. 

There is no prospect of a cure for asthma at the time of writing, but the potential for prevention of asthma by modification of the antenatal and early post-natal environment has been demonstrated. In the Isle of Wight Allergy Prevention Study, a randomised trial where the intervention delivered reduced maternal and infant inhaled and ingested allergen exposure during early development, was associated with a 75% reduction in risk for asthma at 18 years of age ^4^. In the Canadian Asthma Prevention Study, a similar multi-faceted environmental intervention lead to a 50% reduction in asthma to ten years of age ^5^. What is not clear is the mechanism underlying these effective interventions.

For many years, asthma was considered to be a respiratory manifestation of allergy secondary to a mechanism involving aberrant cellular immune responses to common environmental antigens ^6^. This paradigm has now been reconsidered ^7^ and the central role of airway epithelial cells (AEC) in the development of asthma has become a focus of research ^8^. The AEC is the site of initial encounters between inhaled particles and the respiratory system, and is known to be an important component of the innate system and adaptive immunity modulation, with many genes associated with asthma identified from genome-wide association studies that are expressed in the airway epithelium ^9^. Two systematic reviews have described many differences between the characteristics of AEC from children with and without asthma ^10, 11^. One proof-of-concept study described differences in neonatal nasal AEC function for individuals who went on to have asthma symptoms at age four years compared to those who did not ^12^. This study ^12^ also observed that not all individuals with abnormal neonatal AEC function went on to have asthma symptoms, suggesting that asthma requires both abnormal neonatal AEC function and at least one other factor. 

The microbiome of the neonatal airway, i.e., the populations of commensal bacteria in the airway, is also thought to be important to the development of asthma and may be important to the progression from abnormal AEC function to childhood asthma. Nasopharyngeal colonisation within the first 28 days with *M catarrhalis*, *H influenzae* or *S pneumoniae* is associated with a greater subsequent risk of asthma ^13^, and an abnormal mucosal immune response ^14^. Asthma is a complex condition where multiple factors (which could include AEC and lymphocyte function, the microbiome and familial traits) are accepted as being important to the underlying mechanism.

The Breathing Together consortium has come together to consider how AEC function is related to childhood asthma and how this relationship is influenced by lymphocyte function, the microbiome, and genetic variants in early life which ultimately results in abnormal AEC responses to infectious and non-infectious environmental exposures. Our over-arching hypothesis is that epithelial function is abnormal at birth in babies who subsequently develop asthma and progression is driven by abnormal interactions between the epithelium, genetic factors, the developing immune system, and microbial community in the first years of life. 

Our programme of research will also consider how other factors linked to asthma causation ^15^ may be involved in the development of asthma, including exposure to second hand cigarette smoke and respiratory viruses. The purpose of this paper is to describe our key research questions, aims and methodology for our study, called Breathing Together ( http://breathingtogether.co.uk/).

**Key research questions:**

1. How does pulmonary epithelial function evolve from birth to six years in children who develop asthma, and how does the epithelium interact with the evolving immune system and airway microbiome?

2. What are the mechanisms whereby pathogens and aeroallergens modulate epithelial-immune interactions, skewing airway responses towards asthma rather than resolution?

3. Can these pathways be used to identify biomarkers of asthma development, enabling future preventive strategies?

**Our aims are:**

1. To examine evolution of epithelial cell function during the first years of life, and determine the causal relationships with the maturing immune system and airway microbiome.

2. To investigate the molecular mechanisms whereby epithelial-immune-microbial-gene interactions are skewed towards asthma rather than resolution.

3. To identify biomarkers for asthma development, enabling future disease modifying interventions.

### Objectives

1. Recruit newborn babies with detailed antenatal history, and obtain nasal epithelial cells, blood and other samples at five to ten days of age, and follow them longitudinally for three years with serial sampling, retrospectively phenotyping them depending on whether or not they develop recurrent wheeze (see Methods section 2.1)

2. To understand the normal and abnormal developmental interactions between the airway epithelium, the host immune system and the airway microbiome, by comparing the evolving changes in immune and epithelial function in recurrent wheezers from a birth cohort (see Methods section 2.1) with the pathobiology of established mild and severe wheezers (see Methods sections 2.2, 2.3 and 2.4). 

3. Utilize longitudinal data on childhood wheezing and genetics generated from established large UK birth cohorts (STELAR) to determine if the genetic signals in the pathways and molecules identified in objective two correspond to the different epidemiological/genetic wheeze phenotypes identified in STELAR *.*

## Methods

### 1. Programme design

**Figure 1.**
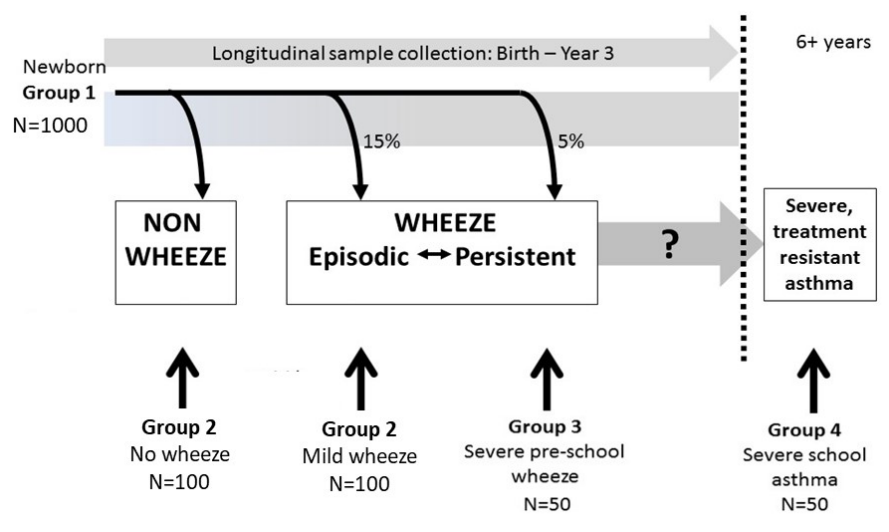
A schematic diagram showing how the participant groups are interlinked.

**Figure 2.**
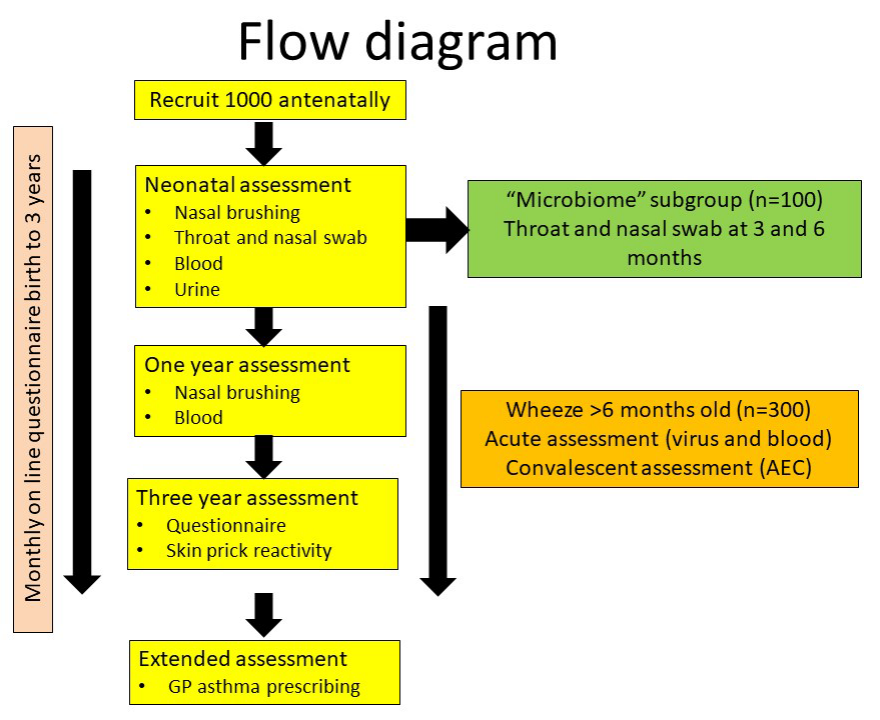
A flow diagram describing the participant’s journey for individuals in participant group one.

The programme design includes four participant groups and five work streams. Figure 1 summarises how the participant groups are integrated. Recruitment to the participant groups began in December 2016 and will continue until Feb 2020. All participants will have nasal AEC collected for culture and transcriptomics, blood or saliva samples collected for DNA analysis, whole blood for RNA transcriptomics, urine sample for cotinine levels and upper airway samples collected for microbiome (see Table 1). Accessing bronchial AEC in healthy neonates is not feasible and nasal AEC will be used as surrogates for bronchial AEC in the neonates we will recruit. Nasal AEC have been shown to be valid surrogates for bronchial cells in five studies involving a total of 124 infants and children ^16– 20^. Bronchial AEC, the gold standard for studying AEC function, will be collected from young children (aged one to ≤ five years) and older children with and without wheeze, when they are under general anaesthetic for a clinically-indicated operation. There are four participant groups:

• Group one (see Figure 2, Methods section 2.1) will consist of a birth cohort of 1000 term singleton neonates where ≥50% have a first-degree family history and from whom nasal AEC will be collected at five to ten days of age and at one and three years thereafter. In total, 100 individuals within participant group one will also have upper airway samples collected for microbiome assessment at three and six months to describe normal colonisation of the upper airways. The remaining 900 individuals in participant group one will form an observational cohort where blood, nasal AEC and nasal secretions will be collected on a wheezy episode after six months of age, with blood being collected in convalescence after six to eight weeks.
• Group two (see Methods section 2.2) will include 200 young children (≤five years) with mild to moderate wheezing, and healthy children with no respiratory symptoms; nasal and bronchial AEC will be collected from participants when they undergo general anaesthetic for a clinically-indicated operation ( Table 1).
• Group three (see Methods section 2.3) will consist of 50 children up to six years of age with severe wheeze undergoing a clinically indicated bronchoscopy. Nasal and bronchial AEC, bronchoalveolar lavage fluid and bronchial biopsy tissue will be collected from participants ( Table 1). Spirometry and exhaled nitric oxide will be measured using standard methodologies ^21, 22^ in participants where possible.
• Group four (see Methods section 2.4) consists of 50 older children with severe asthma undergoing a clinically indicated bronchoscopy. They will be accessed in the same way as participant group three.

**Table 1.** A summary of which samples are collected in each of the four participant groups (PG). mRNA= messenger ribonucleic acid, AEC=airway epithelial cells. *mRNA expression in AEC (but not AEC culture) and lymphocytes also collected at 12 months and three years. ^†^swabs also collected at 12 months and three years of age for all participants and in a subgroup of 100 individuals at three and six months.

	Nasal AEC for culture and mRNA expression	Nasal and throat swabs for microbiome	Urine for cotinine	Blood for lymphocyte mRNA expression	Blood or saliva for DNA	Bronchial AEC for culture and mRNA expression	Bronchial fluid for microbiome	Broncho- alveolar fluid	Bronchial biopsy
PG one (birth cohort)	√ *	√ ^†^	√	√ *	√				
PG two (mild preschool wheeze and control)	√	√	√	√	√	√	√		
PG three (severe preschool wheeze)	√	√	√	√	√	√	√	√	√
PG four (severe asthma in older children)	√	√	√	√	√	√	√	√	√

Lower airway microbiome will be determined in all individuals except those in participant group one.

Ethics approval has been obtained for all aspects of the study (reference numbers 16/LO/1518 [London City and East] and 17/LO/0013 [London-Riverside Research Ethics Committee]). Written informed consent will be obtained from parents and (where appropriate) assent obtained from participants. Material transfer agreements are in place.

### 2 Participant groups

***2.1 Participant group one (birth cohort)***

**2.1.1 Recruitment and eligibility**. This will take place either in the antenatal clinic or on the postnatal ward in five centres (Aberdeen, Edinburgh, London Imperial College, London Queen Mary University Hospital and Isle of Wight). Inclusion criteria will be being born at term (i.e. between 37 and 42 weeks) and having a parent who is able to give written informed consent. There will be no initial active selection for offspring at increased risk for asthma since our earlier work indicates that the nature of this study will likely lead to a participant group which is enriched with individuals with a family history of asthma. Exclusion criteria include: twins or other multiple pregnancies; maternal group B streptococcus on high vaginal swab or urine culture in the present pregnancy (brushing a neonatal nose colonised with this bacterium may lead to invasive infection); need for CPAP or ventilatory support before samples are collected; major concurrent health problems, e.g. congenital heart disease, cystic fibrosis; infants who will not be available to attend acutely if they develop wheeze; infants who are likely to move away from the study centre before three years of age.

**2.1.2 Questionnaires.** The parents will complete a baseline questionnaire to collect data about family history, environment, pregnancy, delivery and early neonatal period ( Supplementary File 1). These data will be collected in the first week of life. Common questions will be used across all participant groups. In addition to baseline questions, a very short online questionnaire will be completed each month by parents until their child is 36 months of age ( Supplementary File 2). To give insight into the natural history of the establishment of airway microbiome for individuals with asthma and without asthma, a subgroup (n=100) will have additional throat and nasal swabs for microbiome analysis at three and six months; a short questionnaire will be completed at the three and six month assessments ( Supplementary File 3). Commercially available software (Qualtrics) will email parents with a link to that month’s questionnaire. Parents will have access to a video of wheeze to provide a standard definition of parental reported wheeze. Where families do not have internet access, researchers will call or text the family on a monthly basis. All participants will be assessed with the same questionnaire at ages one and three years ( Supplementary File 4). This will capture any episodes of wheeze, atopic diseases, any treatment, current environment, nutrition and environment. Questions will be from the International Study of Asthma and Allergy in Children respiratory and eczema modules ^23^ ( Supplementary File 4).

**2.1.3 Acute wheeze assessment.** For participants who are not part of the subgroup (n=100) having additional serial upper airway swabs for microbiome analysis, parents will be invited to contact the team on one (and ideally the first) occasion that their child has an episode of wheeze after 6 months of age (wheeze before this age is likely to be due to infection). A clinical assessment will take place within five days from onset of symptoms. Previously validated questionnaires (Jackson cold score ^24^ and Ducharme pre-school asthma exacerbation questionnaire ^25^) will capture details of symptoms ( Supplementary File 5). These participants will be reassessed six to eight weeks later for a convalescent visit. A convalescent questionnaire will be completed, covering symptoms associated with the acute illness, their current healthcare utilisations and any treatment ( Supplementary File 6). *Clinical assessments*. Participants will be examined by a trained nurse or doctor at the baseline, one year, and three years. The chest will also be assessed for the presence of wheeze at the symptomatic and convalescent assessments and severity of eczema will be assessed using a standard methodology (SCORAD) ^26^ ( Supplementary File 7). Skin prick testing will be undertaken at three years of age with nine common allergens (house dust mite [HDM], grass pollen, tree pollen, cat, dog, alternaria, cows’ milk and egg, peanut and any other clinically relevant allergen; ALK-Abello, Denmark) using the standard methodology ^27^. A mean wheal diameter of ≥3mm will be regarded as positive in the presence of appropriate control responses.

***2.2 Participant group two (mild preschool wheeze and controls)***

**2.2.1 Recruitment and eligibility**. We will recruit young children (aged between twelve months and ≤five years) with a history of mild to moderate wheezing, and those without previous wheezing. Such children do not ordinarily have samples taken from the lower airways in routine practice and to do this purely for research purposes cannot be justified as it requires the use of anaesthesia. We therefore will use blind bronchial brushing ^28, 29^ to collect samples from children who are already undergoing anaesthesia for an elective surgical procedure. Our team is experienced in this methodology ^18, 30^. In addition to being scheduled for elective general anaesthetic, inclusion criteria include age one to ≤five years at the time of sampling and being free from recent respiratory tract infection. Exclusion criteria include: born preterm (i.e. <37 gestation); recent respiratory tract illness (i.e. symptoms resolved for < two weeks); other chronic respiratory illnesses (e.g. Cystic Fibrosis); a history of previous pituitary or ethmoid surgery.

We will recruit 50 children in each of the following four categories:

1. Wheeze and atopy. Recurrent wheezing (more than two episodes of confirmed wheezing with at least one episode in the last six months) and other clinical atopy (e.g., eczema and/or allergic rhinitis).

2. Wheeze and no atopy. Recurrent wheezing (more than two episodes of confirmed ^*^ wheezing with at least one episode in the last six months) and no obvious wheezing triggered by aeroallergens and no clinical evidence of other atopy.

3. No wheeze and atopy. No history of wheezing but with at least one clinically apparent atopic disorder (e.g. eczema and/or allergic rhinitis)

4. No wheeze or atopy. No history of wheezing and with no clinical evidence of other atopic disorder

Wheezing will be confirmed by parents after viewing an audio-video clip of true wheezing.

**2.2.2 Questionnaires.** The same questionnaires used in group one will be used here ( Supplementary File 1 and Supplementary File 4). Respiratory signs and symptoms and including details ^26^ of eczema ( Supplementary File 7) will be documented in participant groups two, three and four.

***2.3 Participant group three (severe pre-school wheeze)***

**2.3.1 Eligibility criteria.** Inclusion criteria for this group include: age twelve months to ≤six years; a history of recurrent episodes of wheeze and difficulty in breathing; due to undergo a clinically indicated bronchoscopy. Exclusion criteria are the same as participant group two (see Methods section 2.1.1).

**2.3.2 Questionnaire.** The same questionnaire will be used for all participant groups ( Supplementary File 1 and Supplementary File 4).

***2.4 Participant group four (severe asthma in older children)***

**2.4.1 Eligibility criteria. ** Inclusion criteria for this group are also described elsewhere ^31^: age six to 16 years; diagnosis of severe asthma made by specialist paediatrician (see Supplementary File 8); documented wheeze by healthcare professional; evidence of one or more of airway hyper-responsiveness confirmed by direct or indirect challenge, bronchodilator reversibility (≥12%) or peak expiratory flow variability of ≥10%. Exclusion criteria include those for participant group two (see section 2.1.1) plus the following: significant alternative diagnoses that may mimic or complicate asthma, in particular dysfunctional breathing, panic attacks, and overt psychosocial problems (if these are thought to be the *major* problem rather than *in addition* to asthma); Poor adherence to therapy demonstrated following a period of electronic monitoring

**2.4.2 Questionnaire.** The same questionnaire will be used for all participant groups ( Supplementary File 1 and Supplementary File 4).

### 3 Collection of samples

Table 1 identifies which samples are collected in each participant group.

***3.1 Nasal brushings for AEC.*** Each nostril will be brushed once with a Dent-o-Care interdental brush 620 (2.7mm brush) ^32^. One brush will be used for airway epithelial cell culture and will be inserted into a sterile 15 ml polypropylene collection tube containing 5ml of DMEM. DMEM medium containing penicillin and streptomycin antibiotics will be added to the polypropylene tube. Samples will be place in a sealed sterile plastic bag and transported, within 72 hours, to the tissue culture laboratory. The second brush (for airway epithelial gene analysis) will be placed into an Eppendorf containing RLT buffer (Qiagen) with 2Mercaptoethanol (ME) added and frozen at -80°C prior to transportation to the laboratory for RNA extraction and transcriptomics analysis.

***3.2 Bronchial brushings for AEC.*** For participant group two, blind brushings will be obtained by passing a sheathed bronchial cytology brush (10-mm disposable cytology brush, BC 202D-2010, Olympus, Southend-on-Sea, Essex, UK) through the endotracheal tube until resistance is felt ^28, 29^. Once resistance is met, the sheath is withdrawn and the brush gently moved with up/down and rotational action before the sheath is returned to its initial position and the brush removed. For participant group three and four bronchial AEC will be obtained under direct vision during bronchoscopy. Briefly, a sheathed bronchial cytology brush is inserted though the working channel of the bronchoscope, the brush is advanced through the protective sheath and gentle brush sampling obtained using an up/down and rotational action, the sheath is replaced and the brush removed. The brush head is then cut off and used for cell culture as described above. This process is repeated to collect a sample for mRNA analysis as described above. 

***3.3 Blood sampling.***For all participant group members, 50 µl whole blood will be collected by Minivette® into specifically prepared tubes with PAXgene Blood RNA reagent. The sample will be stored at –20°C, within 60 mins of collection and will be used for whole genome gene expression arrays in workstream four (see Methods section 4). For participant groups two, three and four, blood will be collected for measurement of (i) total serum IgE, (ii) specific IgE to nine common allergens (HDM, grass pollen, tree pollen, cat, dog, alternaria, cows’ milk and egg, peanut and any other clinically relevant allergen), specific IgE >0.35 is considered positive, and (iii) immune and inflammatory cells in the blood using flow cytometry and genotype.

***3.4 Airway samples for microbiome analysis.*** Separate swabs (ESwab, Becton Dickinson, Wokingham, UK) will be taken from the nose and oropharynx. For individuals in participant groups two, three and four a bronchoscopy cytology brush (10-mm disposable cytology brush, BC 202D-2010, Olympus, Southend-on-Sea, Essex, UK) will be passed into the airways until resistance is felt and then removed, the brush will be cut off and placed in an empty container (Falcon 15ml, Fisher Scientific, Loughborough, UK). The swabs will initially be stored at room temperature until transported back to the local laboratory at the site of collection, where they will be stored at -80°C until transported in batches on dry ice to the laboratory in Monash University for DNA extraction and analysis of the bacterial microbiome by 16S rRNA PCR sequencing. Control containers will also be collected for one in twenty individuals. BAL samples will also be used for assessment of the microbiome. The virome may also be tested using samples collected for microbiome studies and from whole blood RNA.

***3.5 Broncho-alveolar lavage (BAL).*** For participant group two, after intubation the “blind” technique ^33^ will be used and a suction catheter will be passed through the endotracheal tube until it meets resistance, then 1 ml/kg (to a maximum of 20 ml) of normal saline will be instilled via syringe into the catheter, and the fluid will be aspirated back with suction applied. For participants in participant group three and four, BAL will be collected using the bronchoscope ^34^; here three aliquots of 1 ml/kg (to a maximum of 40 ml/aliquot) is passed into the airways after the bronchoscope is wedged and the fluid removed by suction. BAL will be spun, and supernatants stored at -80°C for future analysis of inflammatory mediators. Counts of inflammatory cells, including neutrophils, eosinophils and lymphomononuclear cells will be performed on cell cytospins. Cells will be further characterised by flow cytometry at selected study centres and if sufficient cell numbers are obtained. 

***3.6 Endobronchial biopsies.*** We have shown biopsies can be taken safely for research and without adding significant time to the procedure ^35^. Up to four biopsies will be collected from the 3 ^rd^ or 4 ^th^ generation sub-segmental bronchi. Biopsies will be fixed in formal saline for up to 24 hours and processed to paraffin blocks. Sections will be cut and stained with haematoxylin and eosin to assess overall biopsy quality and morphology. Those containing recognisable epithelium, reticular basement membrane, and associated submucosa will be analysed further for airway inflammation and remodelling.

***3.7 Urine for cotinine.*** Urine will be collected using a bag, and cotinine concentration determined by standard enzyme linked immune assay to objectively measure tobacco smoke exposure in the previous 24–36 hours.

***3.8 Nasopharyngeal aspirate for virology from acute assessment in participant group one*** Samples will be collected (UTM-RT swabs, COPAN, Murrieta, CA) and stored centrally in the Edinburgh laboratory and analysed by (RT)-PCR.

**Figure 3.**
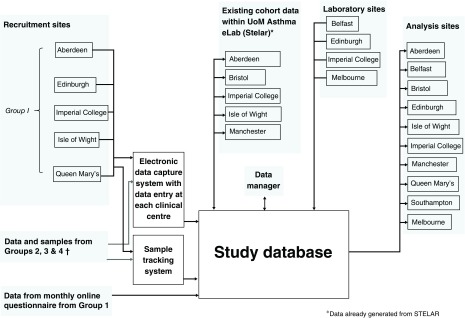
Overview of data management within the project. ^†^recruiting sites for groups 2–4 include Belfast, Southampton, Imperial College, Queen Mary’s and Aberdeen.

***3.9 Data collection and sample tracking*** A bespoke database and electronic case report form (CRF) has been created using InForm® software. This database provides a single portal which captures the responses to all the questionnaires and which allows samples to be identified and tracked. Figure 3 gives an overview of data management within the project. Usual quality control checks are in place, e.g. gender options either male or female, birth weight between 1500 g and 4500 g. Samples from each individual are barcoded and the bar code details linked to the participant’s CRF, and this allows participant details to be linked to samples wherever the samples are analysed.

### 4 Workstreams

There will be five workstreams. The first is the recruitment of participants and collection of samples. The following four workstreams will include a series of experiments that will be conducted using samples collected from participants:

• Workstream 2. Airway epithelial cells. Neonatal cells will be cultured at air liquid interface to achieve a differentiated epithelial cell layer and, as previously ^12^, responses to common environmental exposures will be compared between those who have asthma symptoms and those who have no asthma symptoms by three years of age. RNA will also be extracted from cells and a particular focus will be genes involved in tissue proliferation, repair and remodelling. Differences in responses to environmental exposures and in RNA expression will also be described in children with and without asthma in groups two, three and four.
• Workstream 3. Microbiome. The evolving microbiome characteristics from shortly after birth through to infancy will be described in the 100 individuals where repeated microbiome sampling takes place. Microbiome characteristics shortly after birth and at one and three years will be compared between group one participants who have asthma symptoms and who do not have asthma symptoms at three years of age. In a cross-sectional manner, microbiome characteristics will be described in children with and without asthma in groups two, three and four.
• Workstream 4. Immune system. Whole genome gene expression array will be used to describe RNA expression in lymphocytes and how these change over time and in the context of evolving asthma symptoms by age three years in group one participants. Differences in RNA expression will also be sought between children with and without asthma in groups two, three and four.
• Workstream 5. Genetics. Candidate genes which are known to be involved in tissue proliferation, repair and remodelling will be studied among participants in groups one, two, three and four. Additionally, genetic variants which are found to be of potential relevance to asthma within the STELAR cohorts will be described in the Breathing Together groups.

Many experiments will involve collaboration between workstreams, for example immune system and genetics. State of the art technology, apparatus and analysis will be used in the experiments. In anticipation of their being a limit to the availability of samples, we will have a hierarchy of questions to ensure that the key research questions are addressed whilst also making best use of the samples available to test hypotheses that arise during the course of our experiments.

### 5 Study results dissemination

Study results will be presented at international conferences and published in peer-reviewed journals. Research data will be made available once all data items are cleaned and indexed. We will publish data dictionaries and study metadata (without links to the source data) on the project website for future data sharing on request to the Ethics Committee.

## Summary

The Breathing Together study brings together a multidisciplinary team whose collaboration will yield answers to questions that are crucial to understanding asthma inception. The key outputs of Breathing Together will be to determine (i) whether neonatal AEC function (including response to virus and allergen) is “normal” before the onset of asthma symptoms, (ii) the relationship between the maturing upper airway microbiome and immune system in early infancy and subsequent wheeze, how these are related to changes in AEC RNA pathways, and (iii) how genetic factors modify relationships between AEC and wheeze outcome. 

Recruitment began in December 2016 and Breathing Together teams have been established in Aberdeen, Belfast, Bristol, Edinburgh, Isle of Wight, London (Imperial College and Queen Mary University of London, QMUL), Manchester, Melbourne and Southampton. Samples are being collected and moved between centres for analysis, for example all leukocyte analysis will be undertaken in Edinburgh, Microbiome and AEC RNA analysis will be done in Melbourne, and urinary cotinine in QMUL. The AEC culture experiments will take place in three laboratories (Belfast and both London sites) and transport from Aberdeen, Edinburgh and Southampton has been piloted and proven effective, with cells being cultured and frozen for later analysis. Very clear standard operating procedures are in place between the three AEC laboratories, staff visit other laboratories and samples from the same individuals will be analysed in all three laboratories. 

Obtaining AEC samples from neonates presents a number of practical challenges, and even when these are overcome a small amount of cells are sometimes available which permits only a restricted number of experiments. Our primary AEC exposures will be HDM and respiratory syncytial virus to which cultures from all individuals will be exposed. We will retain AEC from all individuals in anticipation of the birth cohort being followed up beyond three years and novel methodologies and research questions arising in future. 

An important part of Breathing Together is its public engagement. This is being delivered in collaboration with Okido ( http://www.okido.co.uk/), a children’s science magazine which also features in a television programme ( Messy goes to Okido). Okido aim to engage children aged three to five years in science and learning; the additional funding for this is the biggest Public Engagement grant awarded by The Wellcome Trust. 

In short, the Breathing Together team are taking a multidisciplinary approach to deliver cutting edge research in partnership with infants, young children and their parents with an ultimate goal of bringing forward the day when asthma prevention is part of routine antenatal and perinatal practice.

## Ethical statement

Details in Methods (see “Programme design” section 1).

Consent for publication: No patient identifiable data are presented.

## Data availability

No data are associated with this article.

## Supplementary material

Supplementary File 1. Baseline questionnaire completed by participants of birth cohort (group one).

Supplementary File 2. Monthly questionnaire completed by birth cohort participants.

Supplementary File 3. Questionnaire completed at three and six month assessment for 100 individuals recruited to longitudinal microbiome sampling.

Supplementary File 4. Questionnaire completed by all participants. Group one complete this at ages one and three years. Groups two, three and four complete this at recruitment.

Supplementary File 5. Question completed at acute wheeze assessment of the birth cohort.

Supplementary File 6. Question completed at convalescent assessment after an acute wheeze assessment.

Supplementary File 7. Tool for assessment of eczema (from reference 26).

Supplementary File 8. Definition of severe asthma.
