# Supplementary material for: Pulmonary epithelial barrier and immunological functions at birth and in early life - key determinants of the development of asthma? A description of the protocol for the Breathing Together study
Source: Wellcome Open Res. 2018 May 17;3:60. [Version 1] doi: 10.12688/wellcomeopenres.14489.1 (PMC6097397; doi:10.12688/wellcomeopenres.14489.1)
Supplement: Supplementary file 1 [file wellcomeopenres-3-15774-s0000.tgz › e048abaa-f32c-44b6-b2d9-1e1d8eff1820.pdf]

NHS Grampian | breathing together | Imperial College London

Subject ID: ☐☐☐☐ **Visit Date:** ☐☐/☐☐☐/☐☐☐☐

Breathing Together

Day 1

Demographics

Q1. Baby's Full Name ____________________

Q3. Baby's Date of Birth ☐☐/☐☐/☐☐

Q6. Sex (circle) Male / Female

Q7. Ethnicity:

- ☐ White British
- ☐ White Irish
- ☐ Other White
- ☐ Mixed Black & White Caribbean
- ☐ Mixed Black & White African
- ☐ Mixed Asian & White
- ☐ Other Mixed
- ☐ Indian
- ☐ Pakistani
- ☐ Bangladeshi
- ☐ Chinese
- ☐ Other Asian
- ☐ Black Caribbean
- ☐ Black African
- ☐ Other Black
- ☐ Not reported

Any other ethnic group: ____________________

Email address for short online monthly questionnaire

____

Q8. Baby's GP

____

NHS Grampian | breathing together | Imperial College London

Subject ID: ☐☐☐☐ Visit Date: ☐☐/☐☐☐/☐☐☐☐

Family History

Q1. Is there a family history of Asthma? ☐ Yes ☐ No

If 'Yes', please tick all that apply:

- ☐ Baby's Mother
- ☐ Baby's Father
- ☐ Baby's Sister
- ☐ Baby's Brother
- ☐ Baby's half Sister
- ☐ Baby's half Brother

Q2. Is there a family history of Hayfever or Allergic Rhinitis?

If 'Yes', please tick all that apply: ☐ Yes ☐ No

- ☐ Baby's Mother
- ☐ Baby's Father
- ☐ Baby's Sister
- ☐ Baby's Brother
- ☐ Baby's half Sister
- ☐ Baby's half Brother

Q3. Is there a family history of eczema?

If 'Yes', please tick all that apply:

- ☐ Baby's Mother ☐ Yes ☐ No
- ☐ Baby's Father ☐
- ☐ Baby's Sister ☐
- ☐ Baby's Brother ☐
- ☐ Baby's half Sister ☐
- ☐ Baby's half Brother ☐

☐

Subject ID: ☐☐☐☐ Visit Date: ☐☐/☐☐☐/☐☐☐☐

Father's Information

Q1. What is the highest level of education that you completed?

☐Left before GCSE / Standard Grades
☐Completed GCSE Level
☐Completed A-Level / Higher
☐Completed a University Degree
☐ Other (specify)_________________

Q2. Are you currently employed? ☐ Yes ☐ No ☐Unknown

Q2.1 If 'Yes' please specify job title ____________________________

Q3. Do you smoke or have you ever smoked?

☐Yes
☐Yes, ex-smoker
☐No, never smoked

Q4. Do your vape now or have you ever vaped?

☐Yes
☐Yes, ex-vaper
☐No, no never vaped

Q5. Have you ever had wheezing or whistling in your chest at any time in the past? ☐ Yes ☐ No ☐ Unknown

Q5.1 If 'Yes', has this wheezing or whistling been within the <u>last 12 months?</u> ☐ Yes ☐ No ☐ Unknown

Q6. Have you ever had a diagnosis of asthma? ☐ Yes ☐ No ☐ Unknown

Q6.1 If 'Yes', was this diagnosed by a doctor? ☐ Yes ☐ No ☐ Unknown

Q7. Have you ever had a problem with sneezing, a runny nose, or a blocked nose when you did not have a cold or the flu? ☐ Yes ☐ No ☐ Unknown

Q7.1 If 'Yes', have you had these symptoms in the <u>past 12 months</u> when you did not have a cold or the flu? ☐ Yes ☐ No ☐ Unknown

Q8. Have you ever had a diagnosis of hayfever? ☐ Yes ☐ No ☐ Unknown

Q9. Have you ever had a diagnosis of eczema? ☐ Yes ☐ No ☐ Unknown

Q10. Have you ever been diagnosed with a food allergy? ☐ Yes ☐ No ☐ Unknown

Q10.1 If 'Yes', was it diagnosed by a doctor? ☐ Yes ☐ No ☐ Unknown

Subject ID: ☐☐☐☐ Visit Date: ☐☐/☐☐☐/☐☐☐☐

Mother's Information

Q1. What is the highest level of education that you completed?

☐Left before GCSE / Standard Grades
☐Completed GCSE Level
☐Completed A-Level / Higher
☐Completed a University Degree
☐ Other (specify) ________________

Q2. Is the mother currently employed?

☐ Yes ☐ No ☐ Unknown

Q2.1 If 'Yes' please specify job title

____

Q3. Do you smoke, or have you ever smoked?

☐Yes
☐Yes, but ex-smoker
☐No, never smoked

Q3.1 If 'Yes', did you stop smoking, or reduce the number of cigarettes that you smoked when you found out that you were pregnant?

☐Yes, stopped completely
☐Yes, reduced the number of cigarettes
☐No, continued to smoke the same level
☐Ex-smoker who stopped before became pregnant

Q3.2 If 'Ex-Smoker', did the mother stop smoking or reduce the number of cigarettes that she smoked when she found out that she was pregnant?

☐Yes, stopped completely
☐Yes, reduced the number of cigarettes
☐No, continued to smoke the same level
☐Stopped before became pregnant

Q4. Do you now, or have you ever vaped?

☐Yes
☐Yes, but ex-vaper
☐No, never vaped

Q4.1 If 'Yes', did you stop vaping or reduce the amount of vaping when you found out that you were pregnant?

☐Yes, stopped completely
☐Yes, reduced the amount of vaping
☐No, continued to vape at the same level
☐Ex-vaper who stopped before became pregnant

Q5. Were you exposed to passive/second-hand cigarette smoke while you were pregnant?

☐ Yes ☐ No ☐ Unknown

Q6. When you were pregnant, did you take nutritional supplements (such as folic acid, vitamins, fish oil capsules etc)?

☐ Yes ☐ No ☐ Unknown

Subject ID: ☐☐☐☐ Visit Date: ☐☐/☐☐☐/☐☐☐☐

Q6.1 If 'Yes', please specify	____________________
Q7. Have you ever had wheezing or whistling in your chest at any time in the past?	☐ Yes ☐ No ☐ Unknown
Q7.1 If 'Yes', you had wheezing or whistling, was this within the <u>last 12 months?</u>	☐ Yes ☐ No ☐ Unknown
Q8. Have you ever had a diagnosis of asthma?	☐ Yes ☐ No ☐ Unknown
Q8.1 If 'Yes', was this diagnosed by a doctor?	☐ Yes ☐ No ☐ Unknown
Q9. Have you ever had a problem with sneezing, a runny nose, or a blocked nose when you did not have a cold or the flu?	☐ Yes ☐ No ☐ Unknown
Q9.1 If 'Yes' have you had these symptoms in the <u>past 12 months</u> when you did not have a cold or the flu?	☐ Yes ☐ No ☐ Unknown
Q10. Have you ever had a diagnosis of hayfever?	☐ Yes ☐ No ☐ Unknown
Q11. Have you ever had a diagnosis of eczema?	☐ Yes ☐ No ☐ Unknown
Q12. Have you ever been diagnoses with a food allergy?	☐ Yes ☐ No ☐ Unknown
Q12.1 If 'Yes', was it diagnosed by a doctor?	☐ Yes ☐ No ☐ Unknown

Subject ID: ☐☐☐☐ **Visit Date:** ☐☐/☐☐☐/☐☐☐☐

Baby's Birth Details

Q1. Duration of pregnancy (complete weeks) ☐☐

Q2. Did mum need any treatment courses of antibiotics during pregnancy? ☐ Yes ☐ No

If Yes how many ☐

Q3. Baby's birth weight ☐☐☐☐ grammes

Q4. Head circumference at birth ☐☐cm

Q5. Length at birth ☐☐cm

Q6. How was your baby delivered

- ☐ **Normal Vertex**
- ☐ **Normal Breech**
- ☐ **Forceps Delivery**
- ☐ **Vacuum Extraction**
- ☐ **Planned Caesarean Section**
- ☐ **Emergency Caesarean Section**

Q7. Did your baby need to spend any time on the Special Care Baby Unit or Neonatal Intensive Care Unit ☐ Yes ☐ No

NHS Grampian | breathing together | Imperial College London

Subject ID: ☐☐☐☐ **Visit Date:** ☐☐/☐☐☐/☐☐☐☐

Child's Information

Q1. Do you, or did you ever breast feed your baby?

- ☐ **Yes, but not breastfeeding any more.**
- ☐ **Yes still breastfeeding.**
- ☐ **No.**

Q1.1 If 'Yes but not breastfeeding anymore', how old was your baby when you stopped breastfeeding them? ☐☐ days

Q2. Has your baby been required to have any vaccinations? ☐ Yes ☐ No

Q2.2. If yes please give details ______________________

Q3. Has the baby's mother been required to return to work? ☐ Yes ☐ No

Q3.1 If 'Yes', how old was your baby when their mother returned to work? ☐☐days

Q4. Does your baby have any brothers or sisters (including half-siblings)?

- ☐ **Yes**
- ☐ **No**
- ☐ **Unknown**

Q4.1 If 'Yes' how many? ☐☐

Q4.2 If 'Yes' how many are older? ☐☐

Q5. How many people live in the household? ☐☐

Q6. Number of adults ☐☐

Q7. Number of children <18 years ☐☐

Q8. How many bedrooms does your home have? ☐
