# Supplementary material for: Pulmonary epithelial barrier and immunological functions at birth and in early life - key determinants of the development of asthma? A description of the protocol for the Breathing Together study
Source: Wellcome Open Res. 2018 May 17;3:60. [Version 1] doi: 10.12688/wellcomeopenres.14489.1 (PMC6097397; doi:10.12688/wellcomeopenres.14489.1)
Supplement: Supplementary file 2 [file wellcomeopenres-3-15774-s0001.tgz › 6ab3a2ec-f8f0-46cd-8d35-19f75b19ef0d.pdf]

breathing together

Q1 How many time has your child had noisy breathing or whistling in their chest since you last completed this questionnaire?

[ ]

If the answer to this question is zero, respondent goes straight to Q6. If the answer is not zero all questions are to be completed in order Q2 through to Q6

Q2 Has your child had any other symptoms when they were wheezy? Select all that apply:

- ☐ fever
- ☐ cold
- ☐ cough
- ☐ other

If other, please specify:

[ ]

Q3 What treatments has your child received because of their wheezy illness since you last completed this questionnaire? Please select all that apply:

- ☐ blue inhaler: beta-agonist inhaler (e.g. salbutamol, terbutaline, Ventolin, bricanyl)
- ☐ brown inhaler: inhaled corticosteriods (e.g. clenil, budesonide, Becotide)
- ☐ anticholinergic inhaler (e.g. ipratropium, atrovent)
- ☐ oral corticosteroid (e.g. prednisolone)
- ☐ antibiotics
- ☐ other
- ☐ none

If other, please specify:

Q4 Who has treated your child for their wheeze since you last completed this questionnaire?

- [ ] Parents only
- [ ] community nurse
- [ ] general practitioner
- [ ] emergency department
- [ ] outpatients
- [ ] paediatric assessment unit
- [ ] paediatric ward
- [ ] paediatric intensive care unit
- [ ] other

If other, please specify:

Q5 Which of the 4 sound clips is most similar to your child's breathing when they wheeze?

The text, message includes a link to four audio files containing different respiratory noises

- [ ] 1
- [ ] 2
- [ ] 3
- [ ] 4
- [ ] Unsure

Q6 How many courses of antibiotics has your child received since you last completed this questionnaire?
