# Supplementary material for: Pulmonary epithelial barrier and immunological functions at birth and in early life - key determinants of the development of asthma? A description of the protocol for the Breathing Together study
Source: Wellcome Open Res. 2018 May 17;3:60. [Version 1] doi: 10.12688/wellcomeopenres.14489.1 (PMC6097397; doi:10.12688/wellcomeopenres.14489.1)
Supplement: Supplementary file 3 [file wellcomeopenres-3-15774-s0002.tgz › 3301d702-fb5b-401c-8697-417de2e553d2.pdf]

NHS Grampian | breathing together | Imperial College London

Subject ID: ☐☐☐☐ Visit Date: ☐☐/☐☐☐/☐☐☐☐

Breathing Together

Month 3 and Month 6

3 and 6 month additional assessment

Q1. Has your child had any respiratory problems in the last 2 weeks? (cold, chest infection, wheeze, croup) ☐ Yes ☐ No ☐ Unknown

Q1.1 If 'Yes' please specify ____________________________

Q2. Has child had any antibiotics since they were last seen? ☐ Yes ☐ No ☐ Unknown

Q2.1 If 'Yes' Please specify name and their age at the time. ____________________________

Q3. What do you feed your child (select all that apply)?

- ☐ **Breast Milk**
- ☐ **Cow's Milk Formula**
- ☐ **Hypoallergenic formula**
- ☐ **Solids**
- ☐ **Other**

Please Specify: ______________________________

Q4. Comments:

____

____
