# Supplementary material for: Pulmonary epithelial barrier and immunological functions at birth and in early life - key determinants of the development of asthma? A description of the protocol for the Breathing Together study
Source: Wellcome Open Res. 2018 May 17;3:60. [Version 1] doi: 10.12688/wellcomeopenres.14489.1 (PMC6097397; doi:10.12688/wellcomeopenres.14489.1)
Supplement: Supplementary file 4 [file wellcomeopenres-3-15774-s0003.tgz › 003d2d6a-044d-4957-a74d-300a6964911c.pdf]

breathing together

Respiratory Questionnaire

Q1 Has your child ever been diagnosed by a doctor as having bronchiolitis?

- ☐ Yes
- ☐ No
- ☐ Unknown

If yes, how old was your child (in months) at diagnosis?

____

Q2 Has your child ever had wheezing or whistling in their chest?

- ☐ Yes
- ☐ No
- ☐ Unknown

If yes, how old was your child (in months) when it started?

____

Q3 Do you identify any of the 4 sound clips of respiratory sounds as similar to your child's breathing?

- ☐ Yes
- ☐ None
- ☐ Unsure

If yes, which clip: 1, 2, 3, or 4?

____

Q4 How many episodes of wheeze has your child had ever?

- ☐ Zero
- ☐ 1
- ☐ 2
- ☐ 3
- ☐ 4
- ☐ 5
- ☐ 6-10
- ☐ >10

Q5 Breathing Problems since your child was born:

	Yes	No	Unknown
Does your child wheeze with chest infections? (chesty cough and fever)	☐	☐	☐
Has your child wheezed with a cold? (nasal congestion and discharge)	☐	☐	☐
Does your child wheeze in between colds or chest infections?	☐	☐	☐
Has your child wheezed in the last year?	☐	☐	☐
Has your child wheezed in the last 6 months?	☐	☐	☐
Has your child's chest sounded wheezy during or after exercise?	☐	☐	☐
Has your child's wheezing ever been severe enough to limit their speech to only 1 or 2 words at a time between breaths?	☐	☐	☐

Q6 How often has your child's sleep been disturbed due to wheezing?

- ☐ Never
- ☐ less than or equal to 1 night per week
- ☐ more than 1 night per week

Q7 Do the following make your child wheeze?

- ☐ Change of weather
- ☐ Emotion (e.g. excited/upset)
- ☐ Smoky rooms
- ☐ Pollen season
- ☐ Exercise
- ☐ During vacuum cleaning
- ☐ Bed making or dusting
- ☐ Perfume
- ☐ Certain Foods (please specify)
- ☐ Moulds
- ☐ Hairy/furry animals (please specify)
- ☐ Nasal congestions and discharge
- ☐ Other (please specify)

Specify foods, animals, and/or other:

[ ]

Q8 Has a doctor diagnosed asthma in your child?

- ☐ Yes
- ☐ No
- ☐ Unknown

If yes, what age was your child (in months)?

[ ]

Q9 Has your child had any treatment for their breathing?

- ☐ Bronchodilators
- ☐ Inhaled corticosteriods
- ☐ Oral corticosteriods
- ☐ Other
- ☐ No

If other, give details:

[ ]

Q10 If bronchodilators: How old was your child (in months) when it was first given?

[ ]

Q11 If bronchodilators: did it relieve the symptoms?

- ☐ Yes
- ☐ No

Q12 If bronchodilators: How quickly does it relieve the symptoms?

- ☐ in less than 10 minutes
- ☐ longer than 10 minutes

Q13 If bronchodilators: Has your child used some in the last year?

- ☐ Yes
- ☐ No
- ☐ Unknown

Q14 If inhaled corticosteriods: How old was your child (in months) when it was first given?

[ ]

Q15 If inhaled corticosteriods: Has your child been given them in the last year?

- ☐ Yes
- ☐ No
- ☐ Unknown

Q16 If oral corticosteriods: How old was your child (in months) when it was first given?

[ ]

Q17 If oral corticosteriods: How many courses was your child given in the last year?

[ ]

Q18 In the last year, has your child experienced any of the following?

	Yes	No	Unknown
Hospital admissions with asthma/wheeze ?	☐	☐	☐
Visits to emergency department with asthma/wheeze?	☐	☐	☐
Visits to GP or 'out of hours' doctor with asthma/wheeze?	☐	☐	☐
Days off nursery/school due to asthma/wheeze?	☐	☐	☐

Q19 Has your child had a dry cough at night, apart from a cough associated with a cold or chest infection?

- [ ] Yes
- [ ] No
- [ ] Unknown

If yes: How old were they when it started?

[ ]

Q20 Has your child had chest infection/s (chesty cough and fever)?

- [ ] Yes
- [ ] No
- [ ] Unknown

If yes: How many in the last year?

[ ]

Q21 Has your child ever had a rash which was coming and going for at least 6 months? (do not count regular nappy rash)

- [ ] Yes
- [ ] No (Skip to Q25)
- [ ] Unknown (Skip to Q25)

At what age did this rash begin?

[ ]

Q22 Was this rash itchy?

- [ ] Yes
- [ ] No
- [ ] Unknown

Q23 Has your child ever had a rash or eczema that has lasted for at least 7 days or more? (do not count regular nappy rash)

	Yes	No	Unknown
Has your child ever had a rash or eczema that has lasted for at least 7 days or more?	[ ]	[ ]	[ ]
Have any of these rashes at any time affected the fold of the elbows, behind the knees, in front of the ankles, on the cheeks or around the neck, ears or eyes?	[ ]	[ ]	[ ]

Q24 Has you child had this rash in the last year?

- [ ] Yes
- [ ] No
- [ ] Unknown

Q25 Has your child had a problem with sneezing or a runny or blocked nose when they did not have a cold or the flu?

- [ ] Yes
- [ ] No (skip to Q27)
- [ ] Unknown (skip to Q27)

Q26 Has this nose problem been accompanied by itchy-watery eyes?

	Yes	No	Unknown
Has this nose problem been accompanied by itchy-watery eyes?	[ ]	[ ]	[ ]
Has your child had this nose problem in the last year?	[ ]	[ ]	[ ]

Q27 Has your child had an allergic reaction when in contact with animals?

- [ ] Yes
- [ ] No
- [ ] Unknown

Q28 Has you child had colds (nasal congestion and discharge)?

- [ ] Yes
- [ ] No
- [ ] Unknown

If yes, how many have they had in the last year?

[ ]

Q29 Does your child have any adverse reactions to any foods, such as eczema, breathing problems or gastrointestinal problems?

- [ ] Yes (see addtional questions on seperate sheet)
- [ ] No
- [ ] Unknown

Q30 Does your child attend day care?

- [ ] Not appropriate
- [ ] No
- [ ] Childminder
- [ ] Nursery/creche

How old (in months) was your child when s/he first started day care, nursery or school?

[ ]

Q31 What main type of flooring is in the room where your child sleeps?

- [ ] Carpet
- [ ] Wooden
- [ ] Laminate
- [ ] Parquet
- [ ] Linoleum or vinyl tiles
- [ ] Ceramic/terracotta tiles or stone
- [ ] Sea-grass or coir-type matting

Other

[ ]

Q32 Does your child's mattress have an allergy cover?

- [ ] Yes
- [ ] No
- [ ] Unknown

Q33 Do you have any pets at home?

- [ ] Yes
- [ ] No
- [ ] Unsure

Q34 If you have pets at home, please specify where they are allowed:

	Dog	Cat	Other
Not in the house	[ ]	[ ]	[ ]
Not allowed in the bedrooms	[ ]	[ ]	[ ]
Allowed in the bedrooms	[ ]	[ ]	[ ]

Q35 Please list other pets:

[ ]

Q36 Does your child have regular exposure to animals elsewhere?

- [ ] Yes
- [ ] None
- [ ] Unsure

Q37 If yes, which pets?

- [ ] Dog
- [ ] Cat
- [ ] Other

Please list other pets:

[ ]

Q38 How many courses of antibiotics has your child had since they were 12 months old?

[ ]

Q39 Comments:

[ ]
