# Supplementary material for: Pulmonary epithelial barrier and immunological functions at birth and in early life - key determinants of the development of asthma? A description of the protocol for the Breathing Together study
Source: Wellcome Open Res. 2018 May 17;3:60. [Version 1] doi: 10.12688/wellcomeopenres.14489.1 (PMC6097397; doi:10.12688/wellcomeopenres.14489.1)
Supplement: Supplementary file 5 [file wellcomeopenres-3-15774-s0004.tgz › 7f20b275-60ac-422b-aa5b-6a61e32e8379.pdf]

Subject ID: □□□□ **Visit Date:** □□/□□/□□□□

Acute Visit

Breathing Problems Since Your Child Was Born

When did your child become unwell? □□/□□□/□□□□
DD/MMM/YYYY

To what extent do the following symptoms affect your child during the worse 24 hours of their current illness?

**Symptoms**	**0 (No symptoms)**	**1 (Mild symptoms)**	**2 (Moderate symptoms)**	**3 (Severe symptoms)**
Sore Throat	☐	☐	☐	☐
Runny Nose	☐	☐	☐	☐
Sneeze	☐	☐	☐	☐
Nasal Congestion (blocked or stuffy)	☐	☐	☐	☐
Malaise (Tiredness)	☐	☐	☐	☐
Fever (feverish / chills)	☐	☐	☐	☐
Headache	☐	☐	☐	☐
Hoarseness	☐	☐	☐	☐
Earaches	☐	☐	☐	☐
Cough	☐	☐	☐	☐

Subject ID: ☐☐☐☐ Visit Date: ☐☐/☐☐/☐☐☐☐

The following questions help us decide how unwell your child has been.
Please state HOW OFTEN your child has experienced each of the following symptoms during the worse 24 hours of their current illness:

Symptoms	1 (not at all)	2	3	4 (half of the time)	5	6	7 (all of the time)	Cannot answer
Coughing	☐	☐	☐	☐	☐	☐	☐	☐
Wheezing/whistling in the chest	☐	☐	☐	☐	☐	☐	☐	☐
Loud breathing	☐	☐	☐	☐	☐	☐	☐	☐
Fast breathing	☐	☐	☐	☐	☐	☐	☐	☐
Gasping for breath	☐	☐	☐	☐	☐	☐	☐	☐
Stomach pushing out with each breath	☐	☐	☐	☐	☐	☐	☐	☐
Skin pulling in the neck/throat	☐	☐	☐	☐	☐	☐	☐	☐

Please state the DEGREE to which each symptom has been a PROBLEM observed in your child during the worse 24 hours of their current illness

Symptoms	1 (not at all)	2	3	4 (half of the time)	5	6	7 (all of the time)	Cannot answer
Coughing	☐	☐	☐	☐	☐	☐	☐	☐
Sleep disturbed by cough, wheeze or difficulty breathing	☐	☐	☐	☐	☐	☐	☐	☐
Decreased in energy level	☐	☐	☐	☐	☐	☐	☐	☐
Unwilling to move around (e.g. wants to be carried)	☐	☐	☐	☐	☐	☐	☐	☐
Loss of appetite	☐	☐	☐	☐	☐	☐	☐	☐
Requesting more attention and / or care	☐	☐	☐	☐	☐	☐	☐	☐
Irritable / cranky / fussy	☐	☐	☐	☐	☐	☐	☐	☐
Does not respond as well to blue reliever inhaler as usual	☐	☐	☐	☐	☐	☐	☐	☐
Does not respond as rapidly to blue reliever inhaler as usual	☐	☐	☐	☐	☐	☐	☐	☐
The effect of the blue reliever inhaler does not last as long as usual	☐	☐	☐	☐	☐	☐	☐	☐

About the potential cause of your child's wheezing illness:

Might any of the following have caused your child to wheeze on this occasion?

- ☐ Change of weather
- ☐ Emotion (excited / upset)
- ☐ Smoky rooms
- ☐ Pollen Season
- ☐ Exercise
- ☐ During Vacuum cleaning
- ☐ Bed making or dusting
- ☐ Perfume
- ☐ Certain foods
- ☐ Moulds
- ☐ Hairy / furry animals
- ☐ Nasal congestion and discharge
- ☐ Other

Please specify:

☐

About the treatment that your child has received for their current illness:

What treatments has your child received because of their current wheezy illness? Please select all that apply.

- ☐ Beta-agonist inhaler (e.g. salbutamol or terbutaline)
- ☐ Anticholinergic inhaler (e.g. ipatropium)
- ☐ Inhaled corticosteroid
- ☐ Oral corticosteroid (e.g. prednisolone)
- ☐ Antibiotics
- ☐ Other

Please specify:

☐

About the treatment that your child has received for their current illness:

Who has treated your child? Please select all that apply.

- ☐ Parents only
- ☐ Community Nurse
- ☐ General Practitioner
- ☐ Emergency Department
- ☐ Outpatients
- ☐ Paediatric Assessment Unit
- ☐ Paediatric Ward
- ☐ Paediatric Intensive Care Unit
- ☐ Other

Please specify:

Did the parents identify any of the 4 sound clips of added respiratory sounds as similar to their child's breathing?

☐ Yes ☐ No ☐ Unknown

If **yes** which clips:

- ☐ Clip 1
- ☐ Clip 2
- ☐ Clip 3
- ☐ Clip 4

Examination

Q1. Are there any respiratory symptoms? ☐ Yes ☐ No ☐ Unknown

If yes:

Q1.1 Wheeze audible on chest auscultation? ☐ Yes ☐ No ☐ Unknown

Q1.2 Features of upper respiratory tract infections, e.g. nasal discharge, inflamed nostrils, conjunctivitis? ☐ Yes ☐ No ☐ Unknown

Q2. Any sign of Eczema? (if yes, complete SCORAD worksheet) ☐ Yes ☐ No ☐ Unknown

Q3. SCORAD score ☐☐☐ (max 103)
