# Supplementary material for: Pulmonary epithelial barrier and immunological functions at birth and in early life - key determinants of the development of asthma? A description of the protocol for the Breathing Together study
Source: Wellcome Open Res. 2018 May 17;3:60. [Version 1] doi: 10.12688/wellcomeopenres.14489.1 (PMC6097397; doi:10.12688/wellcomeopenres.14489.1)
Supplement: Supplementary file 6 [file wellcomeopenres-3-15774-s0005.tgz › 3d877334-fcf3-49ff-94f1-374b6b09e20c.pdf]

Convalescent Visit

Convalescent Assessment

Has your child recovered?

☐ Yes
How many weeks did it take them to recover?

☐

☐ No
What ongoing problems do they have?

☐ Unknown

About the treatment that your child has received for their current illness:

What treatments has your child received because of their current wheezy illness? Please select all that apply.

☐ Beta-agonist inhaler (e.g. salbutamol or terbutaline)

☐ Anticholinergic inhaler (e.g. ipatropium)

☐ Inhaled corticosteroid

☐ Oral corticosteroid (e.g. prednisolone)

☐ Antibiotics

☐ Other

Please specify:

About the treatment that your child has received for their current illness:

Who has treated your child? Please select all that apply.

- ☐ Parents only
- ☐ Community Nurse
- ☐ General Practitioner
- ☐ Emergency Department
- ☐ Outpatients
- ☐ Paediatric Assessment Unit
- ☐ Paediatric Ward
- ☐ Paediatric Intensive Care Unit
- ☐ Other

Please specify: ☐

Examination

Q1. Are there any respiratory symptoms? ☐ Yes ☐ No ☐ Unknown

If yes:

Q1.1 Wheeze audible on chest auscultation? ☐ Yes ☐ No ☐ Unknown

Q1.2 Features of upper respiratory tract infections, e.g. nasal discharge, inflamed nostrils, conjunctivitis? ☐ Yes ☐ No ☐ Unknown

Q2. Any sign of Eczema? (if yes, complete SCORAD worksheet) ☐ Yes ☐ No ☐ Unknown

Q3. SCORAD score ☐☐☐ (max 103)
