# Supplementary material for: Pulmonary epithelial barrier and immunological functions at birth and in early life - key determinants of the development of asthma? A description of the protocol for the Breathing Together study
Source: Wellcome Open Res. 2018 May 17;3:60. [Version 1] doi: 10.12688/wellcomeopenres.14489.1 (PMC6097397; doi:10.12688/wellcomeopenres.14489.1)
Supplement: Supplementary file 7 [file wellcomeopenres-3-15774-s0006.tgz › c6e1b8f9-37b4-4840-a76d-e4c3b2368576.pdf]

Subject ID: ☐☐☐☐ Visit Date: ☐☐/☐☐/☐☐☐☐

Work Sheet for SCORAD Calculation

Completed by: ____________________________

Section 1: Spread

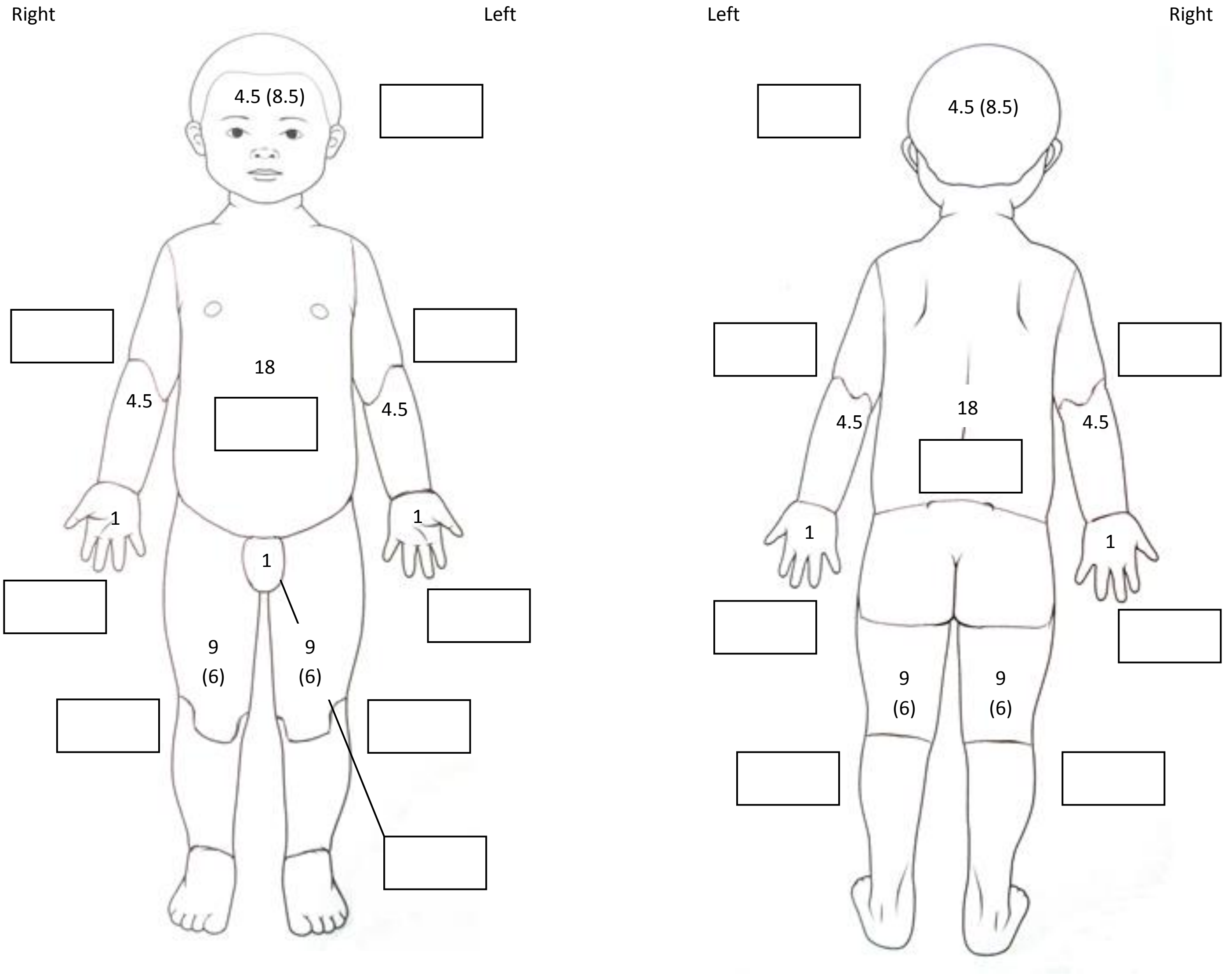

Spread Score (Maximum = 100) ☐ Copy the Spread Score into Section 4.A on the next page.

Continue over the page…

Section 2: Intensity

Criteria	Intensity			
	None (0)	Mild (1)	Moderate (2)	Severe (3)
Redness / Erythma	☐	☐	☐	☐
Oedema / papules	☐	☐	☐	☐
Oozing / crusting	☐	☐	☐	☐
Excoriation	☐	☐	☐	☐
Lichenification	☐	☐	☐	☐
Dryness of unaffected areas	☐	☐	☐	☐

Intensity Score (Maximum = 18) ☐ Copy the Intensity Score into Section 4.B below.

Section 3: Subjective Symptoms

Please mark the **mean score** of the intensity of the following problems over **the last 3 days**, as reported by the primary caretaker.

	0	1	2	3	4	5	6	7	8	9	10
Pruritus	☐	☐	☐	☐	☐	☐	☐	☐	☐	☐	☐
	no pruritus			minimal			moderate				severe

	0	1	2	3	4	5	6	7	8	9	10
Sleeplessness	☐	☐	☐	☐	☐	☐	☐	☐	☐	☐	☐
	sleeps through the night										constantly awake

Subjective Symptoms Score (Maximum = 20) ☐ Copy the Subjective Score into Section 4.C below.

Section 4: SCORAD Score Calculation

4.A – Spread Score (from Section 1): ☐ divide by 5 = ☐

+

4.B – Intensity Score (from Section 2): ☐ multiply by 7 = ☐ divide by 2 = ☐

+

4.C – Subjective Symptoms Score (from Section 3) ☐

=

SCORAD Score (Maximum = 103) ☐
