# Supplementary material for: Pulmonary epithelial barrier and immunological functions at birth and in early life - key determinants of the development of asthma? A description of the protocol for the Breathing Together study
Source: Wellcome Open Res. 2018 May 17;3:60. [Version 1] doi: 10.12688/wellcomeopenres.14489.1 (PMC6097397; doi:10.12688/wellcomeopenres.14489.1)
Supplement: Supplementary file 8 [file wellcomeopenres-3-15774-s0007.tgz › 1a942825-0612-474a-876e-7873b05bfb57.pdf]

Supplement six. Definition of severe asthma

We will include children (aged 6-16 years) who meet the following criteria:

(i) Poor control (as defined below) despite treatment with any inhaled corticosteroid at a dose greater than 800 mcg/day (budesonide) or 500 mcg/day (fluticasone), and long acting β-2 agonists, and current or a previous failed trial of leukotriene receptor antagonists[1].

OR

Need for treatment with any inhaled corticosteroid at a dose greater than 800 mcg/day (budesonide) or 500 mcg/day (fluticasone), and long acting β-2 agonists to maintain control.

OR

(ii) Prescription of maintenance oral corticosteroids other biological agent such as omalizumab, other monoclonal antibody or immunosuppressive treatment whatever the level of asthma control

Poor control is defined as one or more of:

- Persistent chronic symptoms *(most days for >3 months)* or an Asthma Control Test (ACT) or Childhood Asthma Control Test (C-ACT) score of <20
- Persistent airflow obstruction *(FEV_1 <80% post bronchodilator)*
- Recurrent severe exacerbations in the past year *(≥2 per year requiring hospital admission or ≥3 per year requiring high dose OCS for at least 3 days)*
- A single PICU admission in the past year

(iii) ALL children must have an objective assessment of adherence using electronic monitoring, with evidence of ≥80% adherence to maintenance inhaled steroids

REFERENCE

1. Chung KF, Wenzel SE, Brozek JL, Bush A, Castro M, Sterk PJ, Adcock IM, Bateman ED, Bel EH, Bleeker ER, Boulet LP, Brightling C et al. International ERS/ATS guidelines on definition, evaluation and treatment of severe asthma. Eur Respir J 2014; 43: 343–373.
