# Supplementary material for: Pulmonary epithelial barrier and immunological functions at birth and in early life - key determinants of the development of asthma?  A description of the protocol for the Breathing Together study
Source: Wellcome Open Res. 2018 May 17;3:60. [Version 1] doi: 10.12688/wellcomeopenres.14489.1 (PMC6097397; doi:10.12688/wellcomeopenres.14489.1)
Supplement: Peer review history

# Peer review: Referee response for version 1

**DOI:** 10.21956/wellcomeopenres.15774.r33375

**Published:** 2018-07-03

**Authors:** Darryl A Knight

**Affiliations:** 1 Faculty of Health and Medicine and Priority Research Centre for Healthy Lungs, Hunter Medical Research Institute, University of Newcastle, Newcastle, NSW, Australia

The authors are attempting an ambitious, but much needed evaluation of the role of the airway epithelium from birth through to teenage age. The hypothesis that the epithelium is abnormal at birth in babies who go on to develop asthma and driven by abnormal gene environment interactions is exciting and testable given the study design. The addition of Microbiome in upper airways is novel and will certainly contribute to our understanding of its involvement and impact. 

 Some areas require additional explanation and clarification.

 1. Group 2 – text says include 200 young children; Figure 1 says 100. 

 2.1.1: do children need to be first born or does this not matter? 

 Apart from clinical symptoms, There is no mention that I can see of determining atopic status (Rast analysis). Will this be done?  

 Work stream 4: do the candidate genes need to be articulated a priori? How many will be investigated? 

 The one aspect missing from this otherwise excellent study design is an analysis of epigenetic modifications, particularly if looking at longitudinal changes and the impact of exposures. Was this considered?

I have read this submission. I believe that I have an appropriate level of expertise to confirm that it is of an acceptable scientific standard.

# Peer review: Referee response for version 1

**DOI:** 10.21956/wellcomeopenres.15774.r33172

**Published:** 2018-07-02

**Authors:** Franca Rusconi, Elisa Gori

**Affiliations:** 1 Unit of Epidemiology, Anna Meyer Children's University Hospital, Florence, Italy

In this article the Authors describe the protocol for the Breathing Together study on the relationships between abnormal airways epithelium cells, immune cell function and asthma symptoms in the context of early gene-microbiome interactions.

 The study is precisely and comprehensively described also with the help of diagrams. The rationale for the protocol is clearly explained, and sufficient details are presented in the Methods section.

 A few parts/points could benefit from some improvements.

**Introduction:**

 Second paragraph: The Isle of Wight Prevention Study and the Canadian Asthma Prevention Study could only be cited without describing them in detail, which interferes with the flow of reasoning.

 Last paragraph: add “possible” to “asthma causation”.

**Methods 1. Programme design.**

 Are nasal samples safely collected in neonates? Please give details on previous experiences.

 In figure 1 Group 2 and 3 seem to be part of Group 1, while in the text it is not clear how children of Group 2 and 3 are recruited.

 Group one:

- How are the 100 children for microbiome analysis selected?

 

- In order to describe normal colonization of the upper airways, is it enough to study 100 individuals? Please refer to previous experiences.

Group two: please clarify if criteria for inclusion are individuals who undergo general anaesthesia for a clinically-indicated operation and among these, those with a history of wheezing and those without respiratory symptoms. In the text the authors say “will be collected from participants”, and in the Chapter 2.2.1 the authors say “we will recruit young children with a history of mild to moderate wheezing and those without a previous history of wheezing”. Apart from clarification wouldn’t it be simpler to include children already undergoing general anaesthesia in *x *centers, and among these to identify those with such history?

 Groups three and four: please clarify if these children are recruited from all the centers involved in the study.

**Methods. Participant groups three and four.**

 Criteria for exclusion: after bronchoscopy, shouldn’t children with “other” causes of wheezing be excluded, e.g. those with anatomical findings (vascular ring/aberrant vessel etc)?

We have read this submission. We believe that we have an appropriate level of expertise to confirm that it is of an acceptable scientific standard.

# Response

**Published:** 2018-07-02

**Authors:** Steve Turner

**Affiliations:** University of Aberdeen, UK

We are very grateful to Prof Rusconi for her time and comments.

- We will amend the introduction as suggested.
- The 100 microbiome subgroup are chosen without selection for any characteristic, e.g. asthma, atopy or smoking, and we anticipate that this sample size is adequate (acknowledging that the novel nature of our study means that no definitive power calculation is possible). 
- We can clarify that criteria for inclusion for lower airway sampling are individuals who undergo general anaesthesia for a clinically-indicated operation and among these, those with a history of wheezing and those without respiratory symptoms.
- Participants for groups 3 and 4 will be recruited from the two London centres and Southampton. 
- We can confirm that children found to have a congenital airway narrowing at bronchoscopy will not be categorised as wheezers or asthmatics.
